# A calcium sensor – protein kinase signaling module diversified in plants and is retained in all lineages of Bikonta species

**DOI:** 10.1038/srep31645

**Published:** 2016-08-19

**Authors:** Linda Beckmann, Kai H. Edel, Oliver Batistič, Jörg Kudla

**Affiliations:** 1 Institut für Biologie und Biotechnologie der Pflanzen, Universität Münster, Schlossplatz 7, 48149 Münster, Germany; 2 College of Science, King Saud University, Riyadh 11451, Kingdom of Saudi Arabia

## Abstract

Calcium (Ca^2+^) signaling is a universal mechanism of signal transduction and involves Ca^2+^ signal formation and decoding of information by Ca^2+^ binding proteins. Calcineurin B-like proteins (CBLs), which upon Ca^2+^ binding activate CBL-interacting protein kinases (CIPKs) regulate a multitude of physiological processes in plants. Here, we combine phylogenomics and functional analyses to investigate the occurrence and structural conservation of CBL and CIPK proteins in 26 species representing all major clades of eukaryotes. We demonstrate the presence of at least singular CBL-CIPK pairs in representatives of Archaeplastida, Chromalveolates and Excavates and their general absence in Opisthokonta and Amoebozoa. This denotes CBL-CIPK complexes as evolutionary ancient Ca^2+^ signaling modules that likely evolved in the ancestor of all Bikonta. Furthermore, we functionally characterize the CBLs and CIPK from the parabasalid human pathogen *Trichomonas vaginalis*. Our results reveal strict evolutionary conservation of functionally important structural features, preservation of biochemical properties and a remarkable cross-kingdom protein-protein interaction potential between CBLs and CIPKs from *Arabidopsis thaliana* and *T. vaginalis*. Together our findings suggest an ancient evolutionary origin of a functional CBL-CIPK signaling module close to the root of eukaryotic evolution and provide insights into the initial evolution of signaling networks and Ca^2+^ signaling specificity.

Darwin’s often quoted struggle for life in which all organisms try to cope with their surrounding habitat can, to a certain extent, be broken down to a struggle for information^1^. All cells–from unicellular protists to highly specialized neurons in multicellular organisms such as humans–need information processing to fulfill their function. Separation into intra- and extracellular space since the development of cell membranes allowed the formation of a tightly controlled milieu within their cellular boundaries. This compartmentalization enabled cells to create and perceive changes in the concentration of specific molecules. Some of those molecules evolved to widespread second messengers that transmit information by changes in their intracellular concentration^2^.

The function of calcium (Ca^2+^) ions as ubiquitous signaling molecules emerged already in prokaryotes and further expanded early in the evolution of eukaryotes^3^,^4^,^5^,^6^. Spatiotemporally defined changes of intracellular Ca^2+^ concentrations regulate various functions such as gene transcription, metabolism or secretion and are involved in fertilization, development and cell death^2^,^7^. This prevalent function is brought about by the stimulus specificity and the resulting diversity of Ca^2+^ concentration changes within the cell, which are defined by amplitude, frequency, space and time. These concentration changes, designated as Ca^2+^ signatures, are translated at the molecular level by various Ca^2+^ binding proteins (Ca^2+^ sensors) into specific downstream response reactions^8^,^9^,^10^.

Despite the fact, that a tightly controlled Ca^2+^ concentration within the cytosol represents one of the most ancient features of life, the further evolution of the Ca^2+^ signaling toolkit resulted in remarkable differences between major extant clades of the eukaryotes^11^. The evolutionary root of eukaryotic species is hypothesized to be placed between the supergroups of Unikonta, comprising Opisthokonta (including fungi and animals) and Amoebozoa and the supergroup of Bikonta including Archaeplastida (containing plants), Chromalveolates and Excavates^12^,^13^. Comparative evolutionary studies including genomes of a broad range of species on the one hand revealed striking conservation of Ca^2+^ signaling components such as Calmodulin (CaM), but on the other hand also uncovered remarkable divergence especially with regard to Ca^2+^ channels among animals, fungi, plants and many protists^14^,^15^,^16^,^17^. In general, these analyses revealed that many conserved components of the Ca^2+^ signaling machinery arose early in the evolution of eukaryotes, but also that significant diversification occurred during the evolution of animals, fungi, plants and protists. While many such broad evolutionary studies largely focused on Ca^2+^ release components, our understanding on the evolution of Ca^2+^ decoding proteins remained less advanced^6^,^18^.

Calcineurin B-like (CBL) proteins are Ca^2+^ sensor proteins, which are closely related to Calcineurin B (CNB) and to neuronal Ca^2+^ sensor (NCS) proteins. CBLs were first identified in Arabidopsis and obtained their name due to their ability to functionally complement a yeast CNB mutant^19^. As NCS and CNB proteins, CBL proteins harbor four conserved EF-hands arranged in a single globular fold. A distinguishing and defining structural hallmark of CBL proteins is provided by their first EF-hand which in contrast to related Ca^2+^ sensor protein families is composed of 14 amino acids^20^,^21^. Despite this structural aberration, this domain still represents a functional EF-hand in terms of Ca^2+^ binding in which Ca^2+^ is coordinated by six amino acid residues similar to the situation in canonical EF-hands that are composed of 12 amino acids^20^. Membrane targeting, a further structural hallmark of CBL proteins, is brought about by lipid modification. Dual modification by N-myristoylation and S-acylation results in plasma membrane targeting, while multiple S-acylation of the N-terminus results in targeting to the vacuolar membrane^22^,^23^,^24^,^25^,^26^.

In contrast to CNBs, CBL proteins do not constitute a regulatory subunit of a phosphatase holoenzyme but instead specifically form complexes with a family of Serine/Threonine protein kinases. These kinases are most closely related to Sucrose Non Fermenting (SNF) related and cAMP-regulated kinases from yeast and animals and have been designated as CBL-interacting protein kinases (CIPKs). Binding of CBLs to CIPKs usually occurs in a Ca^2+^-dependent manner and is crucial for the activation and targeting of the resulting Ca^2+^ sensor/protein kinase complexes^27^,^28^,^29^,^30^. A highly conserved, unique and defining structural feature of CIPKs is provided by the “NAF domain” which is required and sufficient to mediate the interaction of these kinases with the CBL Ca^2+^ sensor proteins^31^. The name of this highly conserved 21 amino acid containing domain was termed according to the invariance of the amino acids N, A and F^18^,^31^. The NAF domain (Pfam accession: PF03822) forms a hydrophobic helix which interacts with a hydrophobic groove within the CBL proteins^21^,^32^. In higher plants, CBLs and CIPKs form intricate signaling networks that in the dicot Arabidopsis consist of 10 *CBL*s and 26 *CIPK*s while the monocot rice genome, for example encodes 11 *CBLs* and 34 *CIPKs*^18^,^33^,^34^. CBL-CIPK complexes have been reported to regulate a multitude of processes such as K^+^ and NO_3_^−^ uptake, Na^+^ and anion extrusion, and ROS generation and thereby fulfill crucial functions in plant stress adaptation and development^35^,^36^,^37^,^38^,^39^,^40^,^41^. Since their discovery, CBLs and CIPKs were generally assumed to represent plant-specific proteins and, in contrast to Arabidopsis and other higher plants, *CBL* and *CIPK* genes are absent in several green algae lineages such as Chlamydomonas and Volvox. Therefore, it was long assumed that the appearance of CBLs and CIPKs correlated with the evolutionary transition from mobile algae to sessile, multicellular plants^29^. However, sequences encoding for CBL and CIPK proteins were recently identified in the unicellular algal species *Ostreococcus tauri* and *O. lucimarinus* as well as in *Chlorella vulgaris* and *C. spec*^18^,^42^. Remarkably, these studies identified singular *CBL* and *CIPK* genes in the genomes of green algae and monadic numbers of *CBL* and *CIPK* genes in mosses and ferns supporting the notion that the complexity of the CBL-CIPK signaling system concurrently emerged with increasing morphological complexity and enhanced during the evolution of the plant lineage^18^,^23^. Quite surprisingly, *CBL* and *CIPK* related sequences were also recently identified in protozoan, non-plant species such as *Naegleria gruberi* and *Trichomonas vaginalis* raising fundamental questions about the evolutionary origin of this Ca^2+^ sensor-protein kinase signaling module^42^.

Here, we report a combination of phylogenomics and functional analyses investigating the occurrence and structural conservation of CBL and CIPK proteins in 26 species that cover all major clades of extant eukaryotic species. Particularly, our results reveal the presence of at least singular CBL-CIPK pairs in representatives of Archaeplastida, Chromalveolates and Excavates and their general absence in Opisthokonta and Amoebozoa. These findings identify CBL-CIPK complexes as evolutionary ancient Ca^2+^ signaling components that likely evolved in the ancestor of all Bikonta and underwent multiple gene losses in this supergroup and are in line with a hypothesized monophyletic origin of the Bikonta supergroup. Moreover, we identified the complement of *CBLs* and *CIPKs* encoded in the genome of the parabasalid *T. vaginalis* as being most distantly related to the plant representatives of these protein families. Therefore, we used the CBLs and CIPK of *T. vaginalis* to experimentally address their functional and structural conservation. These studies not only reveal strict conservation of structural features such as EF-hand composition and NAF domain presence, but also preservation of biochemical characteristics such as cofactor dependence of the kinase and a remarkable sustainment of the interspecific cross-kingdom protein-protein interaction potential of these Ca^2+^ sensors with their corresponding kinase. Together, our findings reveal an ancient evolutionary origin of a functional CBL-CIPK signaling module and suggest that this ancestral component of the Ca^2+^ signaling machinery likely originated close to the root of early eukaryotic evolution.

## Results and Discussion

### A phylogenomics screen identifies potential CBL proteins in all major Bikonta clades

**Figure 1:**
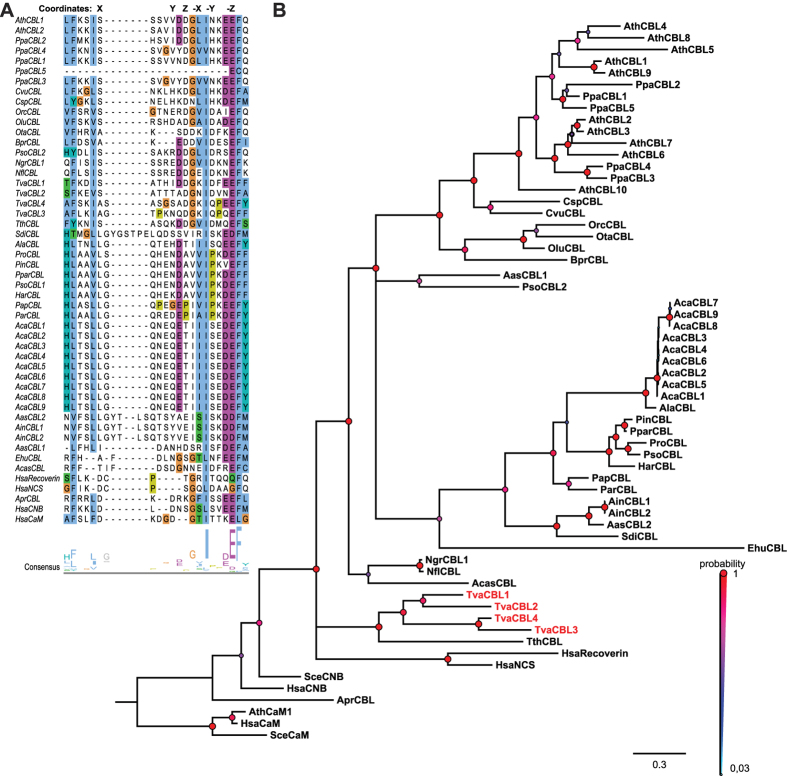
Newly identified Ca^2+^ sensors are phylogenetically closely related to CBLs from *Arabidopsis thaliana* and share characteristic sequence features with CBL-type proteins. (**A**) Multiple Sequence Alignment (MSA) of the first EF-hand of Arabidopsis CBL1 and CBL2 with Ca^2+^ sensors from other species. MSA is visualized by Jalview Version 2 and colored by clustal X color scheme according to conservation (**B**) Phylogenetic relationship of CBL and CBL-related Ca^2+^ sensors. Depicted is a 50%-majority-rule consensus tree based on Bayesian statistics (MrBayes). CBLs and CBL-like candidates cluster on one branch next to their nearest relatives in metazoan (NCS, Recoverin). Values above branches indicate Bayesian posteriori probabilities determined by MrBayes phylogenetic tree inference. The tree was rooted with the CaM branch. Accession numbers are provided in Supplementary Table S1; *Ath: Arabidopsis thaliana, Apr: Auxenochlorella protothecoides, Ain: Aphanomyces invadans, Acas: Acanthamoeba castellanii, Aas: Aphanomyces astaci, Ala: Albugo laibachii, Aca: Albugo candida, Bpr: Bathycoccus prasino, Csp: Chlorella sp., Cvu: Chlorella vulgaris, Ehu: Emiliania huxleyi, Har: Hyaloperonospora arabidopsidis, Ngr: Naegleria gruberi, Nfl: Naegleria fowleri, Ota: Ostreococcus tauri, Olu: Ostreococcus lucimarinus, Orc: Ostreococcus RCC 809, Ppa: Physcomitrella patens, Pin: Phytophthora infestans, Pra: Phytophthora ramorum, Pso: Phytophthora sojae, Pap: Pythium aphanidermatum, Par: Pythium arrhenomanes, Ppar: Phytophthora parasitica, Tth: Tetrahymena thermophila, Tva: Trichomonas vaginalis.*

In order to elucidate the evolution of CBL proteins on a phylogenomics scale, we implemented a multilevel CBL identification strategy. To define an initial CBL-related protein cohort, we screened the genomes of fully sequenced eukaryotes by BLAST analysis using the AtCBL1 (At4g17615) amino acid sequence as a query^43^. With this approach we obtained sequences, which would likely include genuine CBL proteins, but in addition also would represent related Ca^2+^ binding proteins such as NCS or CNB proteins. To further refine our search we performed a phylogenetic analysis based on an M-Coffee multiple sequence alignment^44^,^45^,^46^. This alignment was further analyzed with Bayesian statistics (using MrBayes version 3.2) and is depicted in Fig. 1A as a 50%-majority rule tree^47^,^48^. We included representative, four EF-hand containing, Ca^2+^-binding proteins from human, yeast and Arabidopsis as references. This approach already resulted in the definition of a monophyletic clade that distinguished CNB-NCS-type proteins from other Ca^2+^ sensor proteins such as CaMs. To further percolate for genuine CBL proteins we used the first EF-hand of all candidate proteins as a distinguishing feature^21^. While the canonical EF-hand length of 12 amino acids is absolutely conserved in all known CNB and NCS proteins, this Ca^2+^-binding domain has extended in CBL proteins due to insertion of amino acids between the first (X) and second (Y) Ca^2+^-coordinating amino acids^20^. In addition, in plants the binding coordinate X is mainly occupied by the amino acid S while in the canonical EF-hand the first binding position is always occupied by the amino acid D. Despite these variations, the 14 amino acid EF-hand of CBL proteins represents a functional Ca^2+^-binding domain^20^. A first important result of these combined approaches was that the detailed structural analysis of the first EF-hand strongly supports the results of the phylogenomics calculation. Overall, we newly identified two CBL proteins in unicellular algae species and 25 CBL candidates outside of the Archaeplastida clade. The latter involved 15 species of the Chromalveolates that are representatives of Alveolates, Stramenopiles and Haptophyta. Specifically, CBL sequences were detected for example in *Tetrahymena thermophila*, several species of the genus Phytophthora, two species of the genera Albugo and Aphanomyces and remarkably also in *Emiliania huxleyi*. Moreover, we identified CBL sequences in several species of the Excavates with *N. gruberi* as a representative of Heterolobosea and *T. vaginalis* as a member of the parabasalids. These results provide strong evidence for the occurrence of genuine CBL proteins in all three major clades of Bikonta species.

In sharp contrast, we did not identify any CBL candidate sequence in any species of the Unikonta supergroup. The only potential exception is provided by a *CBL* sequence present in the genome sequence of *Acanthamoeba castellanii,* a representative of the Lobosea. However, the genome analysis of this species revealed that its extant composition was largely shaped by massive lateral gene transfer (LGT) from different species with 465 genes exhibiting best BLASTp values with Excavata species^49^. In agreement with this, the potential AcasCBL sequence identified here most closely groups with the Excavates *N. gruberi* in our phylogenetic analysis (Fig. 1B), suggesting that this gene was transferred into *A. castellanii* by LGT from an Excavata species.

In most of the analyzed algae and non-plant species we identified just one singular *CBL* gene. Notable exceptions were *Aphanomyces invadans* (two genes), *T. vaginalis* (four genes) and *Albugo candida* (nine genes). However, in all these cases our phylogenetic analysis (Fig. 1B) clearly supported intraspecific gene amplification as a reason for the occurrence for multiple *CBL* genes. Overall, the grouping of CBLs within our phylogenetic tree reflected the division of Bikonta into three major phylogenetic lineages (Archaeplastida, Chromalveolates and Excavates)^12^. Analysis of the EF-hand amino acid spacing of the proteins from all species clearly distinguished plant CBL proteins from non-plant CBLs. For example, while the spacer separating EF1 and EF2 is absolutely invariant in land plants and encompasses 23 amino acids, we noticed that this spacing can vary in length from 19 to 24 amino acids in non-plant species (Supplementary Table S1M). In our analyses, CBL proteins encoded in the genome of *T. vaginalis* appeared to be most distantly related to the CBL proteins from plants.

Our detailed inspection of the first EF-hand motif revealed noticeable deviations that likely illustrate evolutionary trends. A first remarkable feature is the absolute invariance of the F residue following the –Z position of the coordinating loop in all CBL, NCS and CNB proteins which is occupied by an L in CaMs (Fig. 1A). Another highly conserved residue is provided by the coordinating X position of the twelve amino acid EF-hand loop which is occupied by a D in NCS, CNB and CaM proteins but provided by an S in genuine CBL proteins. Our analysis supports an ancient origin of this structural feature since this S is conserved in nearly all CBL proteins of the Viridiplantae as well in the CBL proteins of *N. gruberi*, *T. vaginalis* and *T. thermophile*. This supports the notion that an ancient 14 amino acid EF-hand is present in all three major clades of the Bikonta. Remarkably, a further change of the amino acid in this conserved X position appears to coincide with further structural rearrangements of the loop interconnecting the X and Y position and provides a defining feature for monophyletic relations. For example, an S to L exchange was accompanied with an insertion of an additional G into the loop resulting in a 15 amino acid EF-hand and represents a structural motif that is present in all CBLs from Stramenopiles. This deviation of the N-terminal part of the loop interconnecting the X and the Y position coincided with the emergence of a nearly invariant Q-H/N-E motif in the middle part of the loop. Within the Stramenopiles a monophyletic clade including the genera Aphanomyces and Saprolegnia is defined by the addition of at least four further amino acids, which would result in the formation of a 19 amino acid EF-hand structure. Multiple substitutions of conserved amino acids within the first EF-hand of the *E. huxleyi* and *A. castellanii* Ca^2+^-binding proteins coincide with highly accelerated substitution rates of the whole proteins suggesting that both proteins may have lost functionality.

### Concurrent evolution of CBLs and CIPKs in the Bikonta clade

**Figure 2:**
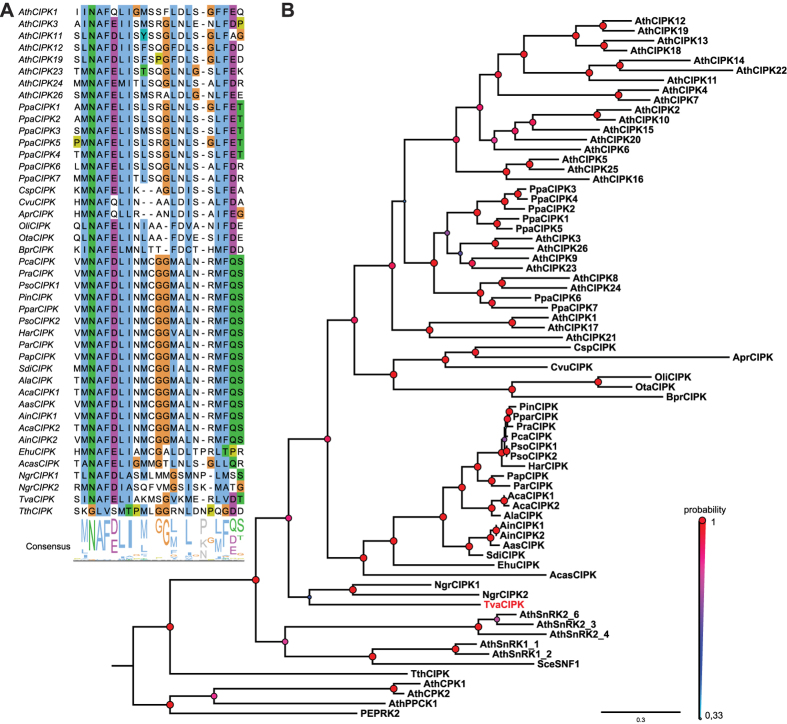
Newly identified CIPKs are phylogenetically closely related to plant CIPKs and contain the CIPK-defining NAF domain. (**A**) MSA of the NAF domain amino acid sequence of Arabidopsis CIPKs, CIPKs from other species and additional related kinases. The characteristic amino acid motif Asn-Ala-Phe (N-A-F) is conserved in all CIPKs but absent in other SNF1-related kinases. Percentage of sequence identity is indicated by colored background (using clustal × color code). (**B**) Phylogenetic relationship of CIPK and CIPK-related kinases. Depicted is a 50%-majority-rule consensus tree derived from Bayesian phylogenetic tree inference. TvCIPK clusters together with all other CIPKs indicating a monophyletic relationship. Values above branches indicate Bayesian posteriori probabilities determined by MrBayes phylogenetic tree inference. Tree was rooted with Yeast SNF1 kinase. Accession numbers are provided in Supplementary Table 1; refer to Fig. 1 for species abbreviations.

A collective attribute of all genuine CBL proteins is their interaction with CIPKs. This interaction mandatorily requires the presence of a highly conserved NAF domain which represents a defining hallmark of this kinase family^18^,^31^. Therefore, we screened the available translated genome sequences of eukaryotic species using a NAF domain Hidden-Markov-Model (HMM; Pfam number PF03822) to identify candidate CIPK sequences^50^. These candidates together with representative related kinases from Arabidopsis (SnRK1s, SnRK2s, CDPKs, PEPRKs) were subsequently aligned using M-Coffee and the resulting MSA employed to calculate a phylogenetic tree based on Bayesian statistics and the MrBayes algorithm^44^,^45^,^46^,^47^,^48^. This phylogenetic tree is depicted in Fig. 2B as a 50%-majority-rule tree. With the exception of *T. thermophile*, this approach identified at least one predicted CIPK sequence in every species that also encoded for CBL proteins. Again, we did not detect a CIPK sequence in any species of the Unikonta supergroup besides in *A. castellanii.* In contrast to the CBL protein from *A. castellanii*, this kinase clustered in our phylogenetic analysis most closely with the representatives from the Chromalveolates. Since the genome of *A. castellanii* contains 628 genes exhibiting highest BLASTp values with Chromalveolates species, we hypothesized that this organism obtained its singular CBL and CIPK sequences in two independent LGT events^49^. It would be interesting to address, if the CBL and the CIPK which are present in *A. castellanii* and likely originated from two different donor species have evolutionarily neo-functionalized into an interacting and functional CBL-CIPK module in *A. castellanii.* Overall, our phylogenetic analysis (Fig. 2B) and detailed inspection of the NAF domain (Fig. 2A) revealed a congruent evolutionary pattern of CBL and CIPK proteins. Quite remarkably, the NAF motif was found to be absolute invariant in all species analyzed. Again, in most non-plant species we detected singular CIPK proteins. The only exceptions with two distinct *CIPK* sequences in the genome were *A. candida* and *A. invadans,* which also exhibit an amplified number of *CBL* genes. Moreover, we detected species-specific CIPK duplications in *P. sojae* and *N. gruberi*. Notably, the CIPK sequence of the algae *A. protothecoides* in which we identified a CNB but no CBL sequence exhibits a highly accelerated substitution rate. This observation would be in agreement with a loss of functionality of this kinase in the absence of an interacting CBL protein. As observed in the evolutionary analysis of CBL proteins, the CIPK protein encoded in the genome of *T. vaginalis* was characterized as being most distantly related to the CIPK proteins from plants.

### *T. vaginalis* expresses interacting CBL-CIPK modules

*T. vaginalis* is an anaerobic, flagellated protozoan and is a sexually transmitted human pathogen causing trichomoniasis, an infection of the urogenital tract^51^. *T. vaginalis* is a representative of the parabasalids within the clade of Excavates^12^,^13^,^52^. Although previously considered to be one of the earliest branching eukaryotic clades, more recent analyses leave the evolutionary relationship of parabasalids to other major eukaryotic groups unclear^12^,^13^. Here, our study characterized the predicted CBLs and CIPK proteins from this species as most distantly related to their plant relatives. Therefore, we chose the CBLs and CIPK from *T. vaginalis* as a model case to assess the functionality of this signaling module. To this end, the coding regions of all four potential CBL proteins were amplified and cloned into BD (binding domain) yeast two-hybrid (Y2H) vectors. Similarly, the *CIPK* and a closely related kinase lacking a NAF domain, here designated as *TvK650* (TVAG_456650), were amplified and cloned into AD (activation domain) Y2H vectors.

**Figure 3:**
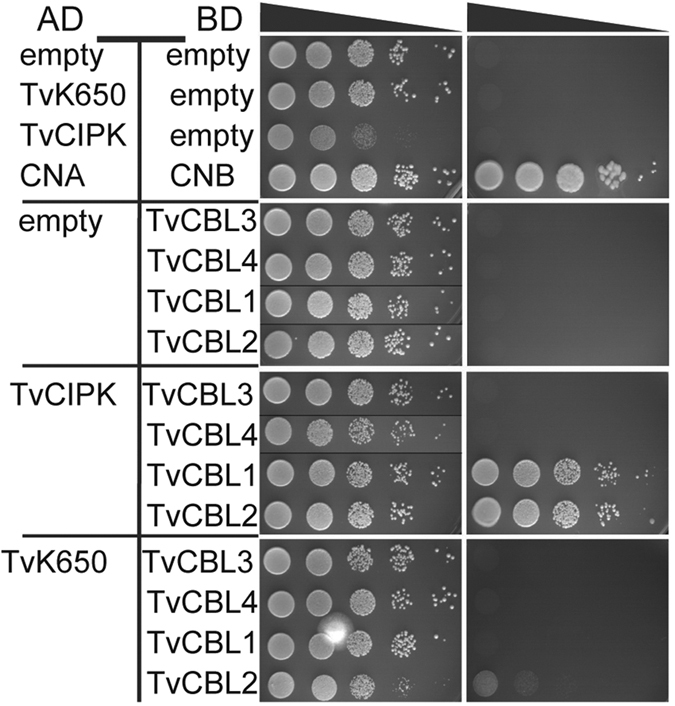
Interaction analyses of TvCIPK and TvCBL1-4 by Y2H assays. TvCIPK interacts with TvCBL1 and 2 but not with TvCBL3 and 4. The interaction exhibits specificity since TvCBL1 and 2 do not interact with the closely related, NAF domain lacking Trichomonas kinase TvK650. Yeast strain (PJ69-4A) containing the indicated pGAD.GH (AD) and pGPT9.BS (BD) plasmids were grown as a dilution series (OD_600_ 10^0^–10^−4^) on SD media lacking Leu (L), Trp (W) (control media; left panel) or L, W, His (H) + 2.5 mM 3AT (selection media; right panel) for 5 days at 23 °C. Growth on SD-LWH media indicates interaction.

To assess potential CBL/CIPK interactions we performed Y2H analyses of different plasmid combinations (Fig. 3). In these experiments *CNA* from rat cloned into the AD plasmid (AD-CNA) and its corresponding *CNB* cloned into the BD plasmid (BD-CNB) were used as positive controls that exhibited robust growth on selective media (Fig. 3 top). In contrast, all combinations of CBL proteins or the two kinases in combination with corresponding empty vectors were not able to grow on selective media. Together, these control experiments establish the selectivity of our assay conditions and their ability to faithfully detect protein-protein interactions. We next combined all four TvCBL constructs with AD-TvCIPK. Of these four combinations only TvCBL1-TvCIPK and TvCBL2-TvCIPK combinations exhibited growth on selective media comparable to the CNA-CNB positive control. In sharp contrast, we did not observe any growth when TvCIPK was combined with either TvCBL3 or TvCBL4. These data identify TvCBL1 and TvCBL2 as being able to efficiently interact with the CIPK protein from the same species. We hypothesize that the loss of their ability to interact with TvCIPK, represents a consequence of the rearranged EF1 in TvCBL3 and TvCBL4. In this regard, both proteins share an S to A substitution in the conserved X position (accompanied with an additional single amino acid insertion–Fig. 1A) which in this form is not observed in any other functional CBL protein. As a final specificity control, we combined all four TvCBLs with the kinase TvK650 that exhibits high similarity with TvCIPK but lacks the NAF domain which has been shown to be essential for interaction of plant CBL-CIPK proteins^31^. These Y2H analyses revealed no detectable growth of any TvCBL-TvK650 combination further supporting the specificity of the identified interactions and suggesting the effective necessity of the NAF domain for establishing sensor-kinase interactions. Finally, we corroborated the functionality of the *T. vaginalis CBL-CIPK* genes by verifying their expression in this species. To this end, RT-PCR analyses were performed with gene specific primers for the two positively interacting CBLs and the CIPK kinase as well as for the *L-lactate/malate dehydrogenase* (86045.m00046) gene that served as a positive control (Supplementary Fig. 1). These experiments clearly supported the expression of these genes in *T. vaginalis*. Together, these results support the ancient origin of CBL-CIPK module formation and point to structural conservation of the underlying mechanisms.

### TvCBL1 and TvCIPK represent a functional Ca^2+^ sensor–kinase module

**Figure 4:**
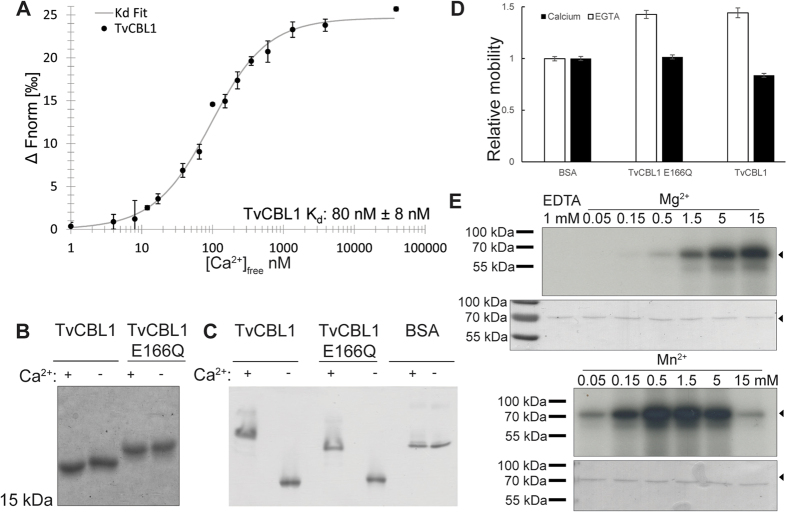
Biochemical characterization of the *T. vaginalis* CBL-CIPK Ca^2+^ sensing module. (**A**) Determination of TvCBL1 Ca^2+^ affinity by microscale thermophoresis. 30 nM TvCBL1 was incubated with a dilution series of Ca^2+^ buffers to determine the K_d_ (**B**) Ca^2+^ binding assay with TvCBL1 and TvCBL1E166Q using SDS-PAGE. 6xHis-tagged TvCBL1 and TvCBL1E166Q protein were incubated with 10 mM CaCl_2_ (+) or 10 mM EGTA (−) and separated by SDS-PAGE. To visualize the mobility shift, the proteins were stained with CBB afterwards. (**C**) Ca^2+^ binding assay with TvCBL1 and TvCBL1E166Q using native-PAGE. (**D**) Mobility quantification of three replicates of the experiment depicted in (**C**) normalized to BSA (EGTA) control. (**E**) Cofactor preference for *in vitro* autophosphorylation of TvCIPK. Upper rows show the autoradiographs of the *in vitro* phosphorylation assays with GST-tagged TvCIPK in the presence of 1 mM EDTA (left), different MgCl_2_ concentrations (upper panel) or different MnSO_4_ concentrations (lower panel). Lower rows show the Coomassie stained TvCIPK. Arrow heads indicate the expected position of TvCIPK protein.

Ca^2+^ binding to their EF-hand motifs represents a fundamental functional feature of CBL proteins for decoding Ca^2+^ signals^53^. In Ca^2+^ sensor proteins, binding of this ion normally induces a conformational change that can be detected by microscale thermophoresis (MST) or by changes of protein mobility in PAGE analysis^54^. The latter approach has been previously used to assess the Ca^2+^-binding ability of plant CBL proteins^19^. To assess the potential Ca^2+^ binding of TvCBL1, we purified recombinant TvCBL1 protein from *E. coli* and analyzed its Ca^2+^ affinity by MST and the mobility of this protein by different PAGE techniques either in the presence or absence of Ca^2+^. As depicted in Fig. 4A MST analyses revealed a high Ca^2+^ affinity with a K_d_ of approximately 80 nM, well within physiological meaningful concentrations. TvCBL1 exhibited accelerated migration in SDS-PAGE and a decreased migration in a native PAGE in the presence of Ca^2+^ indicative for a Ca^2+^-induced conformational rearrangement (Fig. 4B–D). Notably, this effect was more pronounced under non-denaturing conditions. We next sought to address the importance of the EF-hand motifs for Ca^2+^-binding to TvCBL1. For plant CBL proteins, mutations of, for example, the last amino acid in the fourth EF-hand (corresponding to E166Q in TvCBL1) have been reported to strongly diminish Ca^2+^ binding and functionality of these proteins^24^,^35^,^55^. When we comparatively studied the migration of recombinant TvCBL1E166Q protein in the presence or absence of Ca^2+^, we observed a reduced effect of Ca^2+^ on protein mobility when compared to the wild type TvCBL1 protein (Fig. 4B–D). Moreover, the mutated protein exhibited a generally slower migration in the SDS-PAGE that likely resulted from the replacement of the negatively charged E by a positively charged Q. Together, these results establish Ca^2+^ binding and the resulting conformational rearrangements as features of TvCBL1 that enable its function as a Ca^2+^ sensor protein.

We next sought to characterize the biochemical properties of the CIPK protein from *T. vaginalis*. CIPKs from plants, like many other kinases exhibit autophosphorylation activity that often results in enhancing kinase activity towards their targets^36^,^55^. While most kinases require magnesium as a cofactor for enzymatic activity, plant CIPKs have been reported to prefer manganese instead^19^,^36^,^55^,^56^. To assess autophosphorylation activity, we purified recombinant TvCIPK from *E. coli* and performed *in vitro* autophosphorylation assays with radioactive gamma-ATP over a range of concentrations of either Mg^2+^ or Mn^2+^. As depicted in Fig. 4E both ions were able to bring about activation of TvCIPK. However, while it required a minimal concentration of 0.5 mM Mg^2+^ to detect discernable activity of TvCIPK, a concentration of 0.05 mM Mn^2+^ was already sufficient for stronger activation of the kinase. In the presence of Mg^2+^ kinase activity steadily increased up to a concentration of 15 mM Mg^2+^, the highest concentration analyzed here. In contrast, in the presence of Mn^2+^, kinase activity exhibited an optimum in a range from 0.5 mM to 1.5 mM Mn^2+^. Kinase activity was slightly reduced at 5 mM and significantly inhibited at 15 mM Mn^2+^. Remarkably, at a concentration of 0.05 mM Mg^2+^ we did not observe any kinase activity while the same concentration of Mn^2+^ was already sufficient to significantly activate the kinase. Together, these results characterize TvCIPK as an active kinase, which prefers Mn^2+^ ions over Mg^2+^ ions as cofactor. As this is a typical feature of plant CIPKs, which distinguishes this kinase family from many other kinase families, our results suggest that the Mn^2+^ dependence of enzyme activity represents an ancient feature of all CIPKs. Moreover, together with our observation that Ca^2+^ binds to TvCBL1 and that TvCBL1 interacts with TvCIPK, these results support the idea that a functional CBL-CIPK module exists in *T. vaginalis,* which conveys Ca^2+^ signals into downstream phosphorylation responses.

### Cross-kingdom conservation of structural and mechanistic traits required for CBL-CIPK complex formation

**Figure 5:**
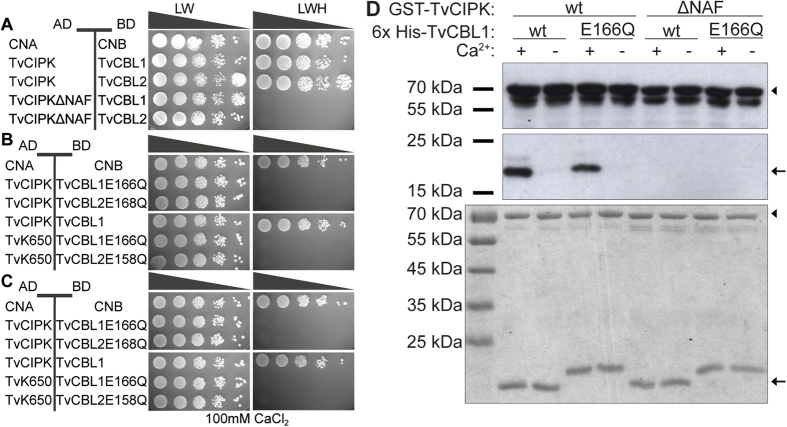
Characterization of TvCIPK–TvCBL interaction. Deletion of the NAF domain in TvCIPK (**A**) as well as mutation of the last EF-hand in TvCBL1 and 2 (**B**) completely abolished the interaction in Y2H assays. Addition of Ca^2+^ to the growth media (**C**) does not rescue the interaction. Yeast strain (PJ69-4A) transformed with the marked plasmids were grown as a dilution series (OD_600_ 10^0^–10^−4^ diluted with 2% glucose solution) on SD media lacking L and W or L, W and H + 2.5 mM 3AT for 14 days (7 days in case of ΔNAF) at 23 °C. (**D**) *In vitro* interaction analyses in presence and absence of Ca^2+^-ions. GST-tagged TvCIPK and -TvCIPKΔNAF were used as bait-proteins and detected with α-GST antibody in each lane (upper panel). 6xHis-tagged TvCBL1 and -TvCBL1E166Q were used as prey-proteins and only detected with α-penta His antibody when interacting with the respective bait-proteins (middle panel). 0.2 mM CaCl_2_ (+) or 0.2 mM EDTA (−) were used to analyze the Ca^2+^-dependency of the interaction. On a third SDS-gel (lower panel), the protein-combinations used here, were separated without performing the interaction analysis and the proteins were stained with CBB to show equal amounts of used protein. Arrow heads indicate the expected position of TvCIPK protein and arrows indicate the position of CBL proteins.

To address the mechanistic principles and evolutionary conservation that enable CBL-CIPK interaction we first examined the role of the NAF domain within TvCIPK for its interaction with TvCBLs. To this end, we performed Y2H analyses as well as *in vitro* interaction analyses. When TvCIPKΔNAF, which lacks the NAF domain, was combined with TvCBL1 or TvCBL2 in Y2H assays, efficient growth was observed only on media that were selective for the presence of both plasmids (LW) but not on media lacking LWH that were selective to detect protein-protein interactions (Fig. 5A). Similarly, when GST-TvCIPK wildtype (Wt) protein and GST-TvCIPKΔNAF protein were combined with His-TvCBL1 in *in vitro* interaction analyses, an absolute requirement of the NAF domain for the interaction of the kinase with the Ca^2+^ sensor was observed. While TvCIPK Wt efficiently interacted with TvCBL1, no interaction of the Ca^2+^ sensor with TvCIPKΔNAF was detected (Fig. 5D). The presence of all proteins in these assays was verified by Coomassie Brilliant Blue (CBB) staining (Fig. 5D). Remarkably, in these *in vitro* analyses we also observed a strict Ca^2+^ dependence for the interaction of TvCBL1 with TvCIPK. While an efficient interaction was observed in the presence of 0.2 mM Ca^2+^, chelation of Ca^2+^ in the assay buffer completely abolished interaction of the Ca^2+^ sensor with the kinase. To further analyze this Ca^2+^ dependence, we replaced the Ca^2+^ coordinating E166 in the fourth EF-hand of TvCBL1 and TvCBL2 by Q and investigated the consequences of this mutation comparatively in Y2H analyses. The interaction of TvCIPK with TvCBL1E166Q was tested *in vitro* as well. The mutation of the fourth EF-hand in TvCBL1 and TvCBL2 completely abolished the interaction with TvCIPK in Y2H analyses and this interaction could not be restored by supplementation of 100 mM CaCl_2_ to the growth medium (Fig. 5C,D). In our *in vitro* interaction analyses, we observed a slight reduction of CBL-CIPK interaction as a consequence of the E166Q mutation when compared to the Wt TvCBL1 protein (Fig. 5D). Again, this interaction was completely abolished by chelation of Ca^2+^ from the assay buffer. These results unambiguously establish Ca^2+^ dependence for the interaction of CBL1 and CBL2 with the CIPK from *T. vaginalis.* Together, the findings of our interaction analyses in yeast and *in vitro* reveal a striking conservation of fundamental features of CBL-CIPK complex formation and an ancient origin of its Ca^2+^ dependence. Studies on plant CBL-CIPK complex formation have revealed the absolute requirement of the NAF domain within the kinase for its interaction with CBL proteins for all CBL-CIPK combinations that have been experimentally analyzed^31^,^36^. The similar essential structural requirement for formation of CBL-CIPK complexes from *T. vaginalis* indicates that the inclusion of the NAF domain in the CIPK kinase protein represents an early evolutionary event that enabled the formation of this two-component signaling module. Since the NAF domain is highly conserved and present in all previously characterized and here newly identified CIPKs, but absent in all other extant kinases, this structural motif is a defining and unique feature for the existence of CBL-CIPK mediated signal transduction. Our results reported here indicate a strict Ca^2+^ dependence for the interaction of TvCBL1/2 and TvCIPK. Similarly, a strict Ca^2+^ dependence was reported for the formation of AtCBL1–AtCIPK1 complexes from *A. thaliana*^27^. In contrast, another Ca^2+^ sensor from Arabidopsis, namely CBL4, has been reported to Ca^2+^ independently interact with the kinase CIPK24 from this species^57^. This situation makes it tempting to speculate that Ca^2+^ dependence of CBL-CIPK interaction represents an ancient feature of this signaling module, which is encoded by singular *CBL* and *CIPK* genes in algae and non-plant species. However, the amplification of multiple *CBL* and *CIPK* genes during the evolution of the Viridiplantae likely allowed for a modulation of this Ca^2+^ dependence. This feature of the plant CBL-CIPK signaling systems clearly deserves further analyses in the future. Nevertheless, a variation of Ca^2+^ dependence of distinct CBL-CIPK pairs may represent a mechanism to fine-tune and to allow for the existence of multiple complex Ca^2+^ signaling processes in higher plants.

**Figure 6:**
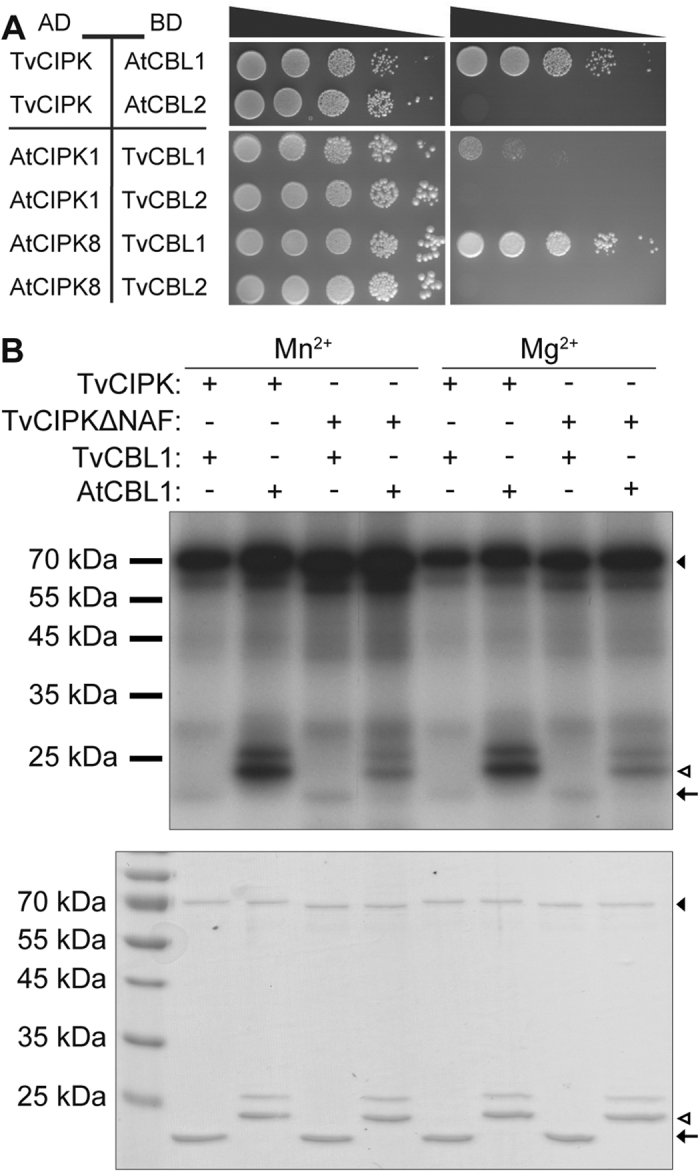
Interaction ability and specificity is conserved between Trichomonas and Arabidopsis CBL-CIPK networks. (**A**) TvCIPK cloned in pGAD.GH tested with AtCBLs cloned in pGPT9.BS shows interaction with AtCBL1. TvCBL1 and 2 tested against AtCIPK1 and 8 only show interaction with AtCIPK8. Drop tests were grown for 14 days (7 days in case of TvCIPK) on SD-LW and SD-LWH + 2.5 mM 3AT media at 23 °C. (**B**) *In vitro* phosphorylation of TvCBL1 and AtCBL1 by TvCIPK. Phosphorylation of 6xHis-tagged TvCBL1 and StrepII-tagged AtCBL1 by GST-tagged TvCIPK and -TvCIPKΔNAF was analyzed in the presence of 0.5 mM MnSO_4_ or 15 mM MgCl_2_, respectively. Upper panel shows the autoradiograph of the *in vitro* phosphorylation assay. Lower panel shows the Coomassie-stained proteins. Arrow heads indicate the expected position of TvCIPK protein and black arrows indicate the position of TvCBL1 proteins and white arrows heads indicate position of AtCBL1.

We next were interested in investigating to which extent the intraspecific evolutionary selection to maintain the ability for CBL-CIPK interaction necessarily results in an interspecific ability of CBL-CIPK interaction over a large evolutionary range of species. To address this question, we chose AtCBL1 and AtCBL2 as models. AtCBL1 and TvCBL1 share an N-terminal MGXXXS/T myristoylation motif that is essential for plasma membrane localization of this protein in Arabidopsis^22^,^35^. In contrast, AtCBL2 does not harbor this motif and is targeted to the vacuolar membrane by palmitoylation of multiple C residues in its N-terminal domain^25^. When we combined the CBL proteins from Arabidopsis with TvCIPK in Y2H analyses, we observed efficient interaction of AtCBL1 with TvCIPK, but no interaction of AtCBL2 with the kinase from *T. vaginalis* (Fig. 6A). We next combined the two CBLs from *T. vaginalis* (TvCBL1 and TvCBL2) with two representative CIPKs from Arabidopsis (AtCIPK1 and AtCIPK8) in similar analyses. AtCIPK1 and AtCIPK8 were used since they belong to structural different subgroups of CIPKs and show distinct CBL interaction patterns also with regard to the Ca^2+^ dependence of the interaction^31^,^32^,^36^. In these experiments, TvCBL1 efficiently interacted with AtCIPK8 and to some extent also with AtCIPK1. In contrast, TvCBL2 did not interact with any of the two kinases (Fig. 6A). These results indicate that in principle the ability of CBLs and CIPKs to interact has remained conserved during the separate evolution of Archaeplastida and Excavates. Within the Viridiplantae, the myristoylation-dependently plasma membrane localized CBL1-type Ca^2+^ sensors represent the primordial kind of Ca^2+^ sensor from which during the evolution of mosses the tonoplast localized CBL2-type Ca^2+^ sensors derived^18^,^58^. Here, we observed interaction of TvCIPK with the ancient CBL1-type CBL from Arabidopsis but not with the derived AtCBL2. This observation is in line with a scenario in which multiplication of a former unique gene allows for evolutionary adaptation to new functions (and cellular protein localizations). In this case, the structural constraints resulting from the selection pressure for upholding CBL-CIPK interaction could be released by simultaneous multiplication of the formally unique *CIPK* gene. Exactly this scenario occurred during the evolution of the CBL-CIPK signaling network within the Viridiplantae. As a consequence, sequence coevolution in interacting CBL-CIPK pairs not only allowed for neo-functionalization of newly formed CBL-CIPK pairs but also provided the basis for the specificity of CBL-CIPK complex formation that is observed in extant higher plants^24^,^31^,^55^. Accordingly, as observed here, the derived AtCBL2 can specifically interact with several CIPKs from Arabidopsis, but lost its ability to interact with TvCIPK^31^. The genome of *T. vaginalis* encodes only one CIPK but four CBL-type Ca^2+^ sensors of which two (TvCBL1 and TvCBL2) retained the ability to interact with TvCIPK. In our interaction analyses, we observed that both TvCBL1 and TvCBL2 were able to efficiently interact with TvCIPK but only TvCBL1 was able to interact with the two kinases from Arabidopsis that were studied here. Again this result supports the conclusion that on one hand the potential for CBL-CIPK interaction can be conserved over long evolutionary distances but also that gene duplication releases the structural constraints underlying CBL-CIPK interactions. This suggests that simultaneous coevolution of specifically interacting CBL-CIPK pairs can create structural solutions that still allow for the interaction of this specific protein pair but prevent interactions with additional members of this protein family. In this way, the evolution of the CBL-CIPK system provides an example for the development of mechanistic principles that underlie the generation of specificity in signal-response-coupling of complex signaling networks in biological systems. In any case, our study identifies *T. vaginalis* as a most interesting evolutionary snapshot in which one partner (CBL) of a formally unical two-component system already underwent amplification but the lack of amplification of the second partner (CIPK) still precludes the formation of novel specific interacting CBL-CIPK modules. In this way, *T. vaginalis* may represent an intermediate evolutionary status in the transition of a singular two-component signaling module into a complex signaling network. The potential further evolution is then exemplified within the Viridiplantae linage where the liverwort *Marchantia polymorpha* for example has 2 *CIPKs* and 3 *CBLs*, the moss *P. patens* harbors 5 *CBLs* and 7 *CIPKs* and finally the genome of *A. thaliana* encodes 10 *CBLs* and 26 *CIPKs*^18^,^33^,^34^.

Previous studies revealed that in Arabidopsis phosphorylation of CBLs by their interacting kinases represents a general feature of these signaling modules and is required for full activity of CBL-CIPK complexes towards their substrate proteins^55^,^59^. CBL phosphorylation usually occurs within a conserved FPSF amino acid motif close to the C-terminus of these Ca^2+^ sensor proteins^55^,^59^. In the work reported here, we noticed that all CBL proteins outside of the Viridiplantae lineage have a shorter C-terminal domain or in the case of the Stramenopiles clade display no discernable FPSF motif in their C-Terminus. This raised the question whether CIPK mediated phosphorylation of CBL Ca^2+^ sensors specifically emerged during the evolution of the Viridiplantae lineage or if alternative amino acid motifs in the CBL proteins from non-plant species may be subject to this regulatory protein modification. To address this facet of CBL-CIPK regulation, we investigated the potential phosphorylation of recombinant TvCBL1 protein by TvCIPK *in vitro.* In these analyses, we also included AtCBL1 since our studies identified this Ca^2+^ sensor from Arabidopsis as interacting with TvCIPK and because previous studies had established efficient phosphorylation of AtCBL1 by AtCIPK1^55^. Furthermore, as an additional control we studied TvCIPKΔNAF as a mutated kinase version that is not able to interact with CBL proteins. *In vitro* phosphorylation assays were comparatively performed in the presence of 0.5 mM Mn^2+^ or 15 mM Mg^2+^. As depicted in Fig. 6B, TvCIPK did not phosphorylate TvCBL1. In contrast, the full-length kinase from *T. vaginalis* efficiently phosphorylated AtCBL1. However, contrary to the full-length kinase protein, deletion of the NAF domain from TvCIPK abolished its ability to phosphorylate AtCBL1. These results suggest, that CIPKs from non-plant species already possess the ability to phosphorylate an FPSF motif containing substrate. Moreover, the mechanism of CIPK mediated CBL phosphorylation requires efficient CBL-CIPK complex formation via the NAF domain of the kinases. These results also suggest, that at least the CBL proteins from *T. vaginalis* do not contain a Ser/Thr residue that is phosphorylatable by a CIPK. These observations are in line with a scenario in which a preexisting ability of CIPKs to phosphorylate an FPSF motif was adopted as a regulatory mechanism for further fine-tuning the activity of CBL-CIPK complexes during the evolution of Viridiplantae.

### Implications of CBL-CIPK evolution

**Figure 7:**
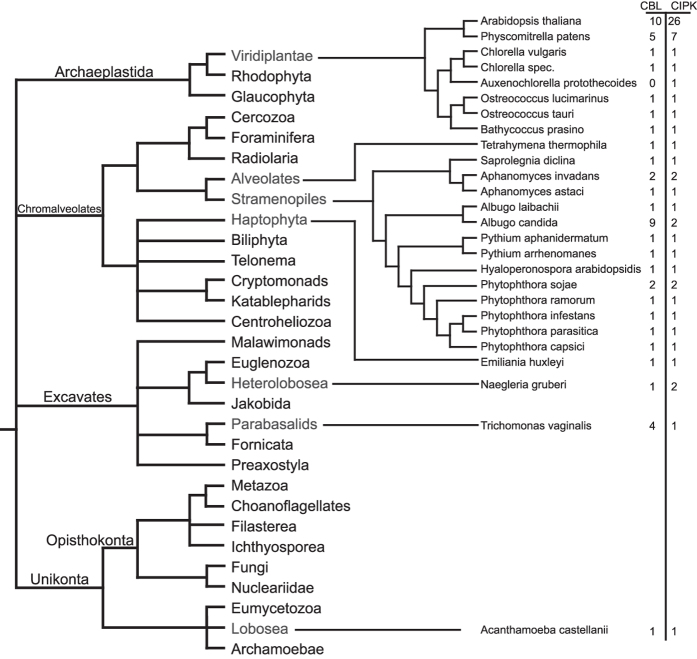
The CBL-CIPK Ca^2+^ sensing module across the eukaryotic tree. The basic eukaryotic tree is depicted according to the tree of life web project (http://tolweb.org). Additionally, phylogenetic relations and absolute number of CBL/CIPKs of the individual species are given.

It is generally assumed, that ancestral eukaryotes diversified into two major lineages, the Unikonta (including the vertebrates) and the Bikonta (including plants)^12^,^13^. Bikonta represent a phylogenetic supergroup divided into Excavates, Chromalveolates and Archaeplastida that encompasses a huge variation of organisms ranging from minute single cell protists to gigantic trees that can live for several thousand years such as *Sequoiadendron giganteum.* Nevertheless, the basic principles of Ca^2+^ signaling appear to be universal in all eukaryotes^4^,^6^,^60^,^61^. Several studies also supported the notion that significant differences in the Ca^2+^ signaling machinery are found among distinct groups of organisms such as animals, fungi and plants^15^,^62^,^63^,^64^. In this regard, it has been suggested, that in contrast to the complexity of the Ca^2+^ signaling toolkit in animals, plants and fungi have evolved a more simplified Ca^2+^ signaling machinery^6^,^63^,^65^. However, our results here provide an example that the Ca^2+^ signal decoding toolkit in the plant lineage has evolved a degree of complexity that is not found in animals. This may reflect fundamental functional and evolutionary differences in the specification of Ca^2+^ signaling in animals and plants. Most of the available studies focused on components that are crucial for Ca^2+^ release or extrusion from the cytoplasm^11^,^15^,^66^,^67^,^68^. In contrast, evolutionary analyses of Ca^2+^ decoding components are less common but provide valuable insights into the evolution of Ca^2+^ signaling^4^,^18^,^69^. Our analysis of the evolutionary distribution of the CBL-CIPK system identified this bimodular Ca^2+^ signaling tool in representatives of all three major Bikonta lineages (Fig. 7). Within the Archaeplastida *CBL* and *CIPK* genes appear to be only present in the Viridiplantae but absent in Rhodophyta and Glaucophyta (Fig. 7). Within the Chromalveolates, we detected CBL-CIPK pairs only in Alveolates and Stramenopiles and in the more distantly related *Emiliania huxleyi* as a representative of the Haptophyta. Similarly, within the Excavates CBLs and CIPKs appear to be sporadically distributed and were found only in Heterolobosea and in *T. vaginalis* as a representative of the parabasalids. We discussed already above, that our identification of a CBL and a CIPK in the Unikonta species *A. castellanii* most likely results from LGT. This may raise the question whether the erratic distribution of CBL-CIPK pairs rather results from LGT instead of following from an ancient evolutionary origin of this signaling system at the basis of Bikonta evolution. However, we consider LGT as a rather unlikely scenario. This is based on several findings. For example, our evolutionary analysis of the EF1-hand sequence of CBLs clearly supports a consecutive order of evolutionary events that shaped the sequence of the extant EF-hands in different species. Moreover, if LGT would have occurred, one would assume that as a consequence the transferred genes exhibit plant-like features. This is clearly not the case for the CBLs. Here, for example the amino acid spacing of the EF-hands as well as the plasma membrane targeting sequence which contains a conserved palmitoylated cysteine in the third position of the protein in all plasma membrane targeted plant CBLs, clearly distinguish plant CBLs from all other CBL proteins identified in this study (Supplementary Table S1.) Finally, the phylogenetic reconstructions of CBL and CIPK evolution reported here is overall in agreement with the current model of the Bikonta evolution (Figs 1B, 2B and 7).

Our findings of course raise the question about the ancient function of CBLs and CIPKs in Ca^2+^ signaling. Ca^2+^ regulation has been shown to be implemented in processes such as life cycle, motility and pathogenicity^66^,^70^. In this regard, our observation that the CBL-CIPK interaction in *T. vaginalis* appears to be Ca^2+^ dependent, supports the notion that this CBL-CIPK pair is indeed linked to Ca^2+^ regulated processes. Quite remarkably, most of the protist (non-plant) species in which we newly identified CBL-CIPK pairs have been described as pathogens for animals or plants. This makes it tempting to hypothesize that CBL-CIPK function in these species may be linked to their pathogenic lifestyle. In this regard, it is for example well established that Ca^2+^ influences trichomonad recognition and binding to host fibronectin during infection^71^. Moreover, one of the *T. vaginalis CBLs* (TvCBL4) was found to be more strongly expressed in high pathogenic isolates of *T. vaginalis* compared to low virulent strains^72^. Again, this could point to a function of Ca^2+^ and CBL-CIPK signal transduction in the infection pathway of this parasite. Finally, our study revealed several examples of evolutionary independent amplifications of either *CBLs* (with four *CBLs* in *T. vaginalis*), or *CIPKs* (with two *CIPKs* in *N. gruberi*) or both (as found in *P. sojae, A. candida* and *A. invadans*). The CBL-CIPK module underwent extensive amplification during the evolution of Viridiplantae and nowadays fulfills many diverse and important functions in plants^18^,^30^. The different states of transition into forming a multi-component signaling network that we identified here may therefore represent interesting snapshots of signaling network evolution. In this regard, the CBLs and CIPKs from *T. vaginalis* and the other species can provide useful subjects to study and reconstruct the initial evolution of complex signaling systems and establishment of signaling specificity in the decoding of the universal Ca^2+^ signal.

## Materials and Methods

### Cloning of *T. vaginalis* CBLs and CIPKs

Molecular biology work was performed according to published protocols^73^. The open reading frames of TvCIPK, TvK650 and the TvCBLs 1, 2, 3 and 4 were PCR amplified from *T. vaginalis* genomic DNA (kind gift of Katrin Henze and Silke Rosnowsky, University of Düsseldorf, Germany) using Pwo Polymerase (Peqlab). Primers used for PCRs are given in the Supplementary Table S2. After restriction digestion the PCR products were inserted into the yeast vectors pGAD.GH and pGPT9.BS, respectively^31^. All constructs generated were verified by sequencing.

Sequence mutations (TvCBL1E166Q and TvCBLE168Q) were introduced by the QuikChange^®^ Site-Directed Mutagenesis Kit from Stratagene. Sequence deletion (TvCIPK∆NAF) was introduced according to^74^. Primers to introduce mutations are given in Supplementary Table S2.

### Bioinformatics analyses

Amino acid sequences of potential CBL proteins from the different organisms were obtained by genome BLASTp analysis using the Arabidopsis CBL1 amino acid sequence as a bait^43^. These BLASTp searches were performed using different databases (**JGI Genome Portal** (http://genome.jgi.doe.gov/; *Naegleria gruberi v1.0; Phytophthora sojae v3.0; Phytophthora ramorum v1.1; Chlorella variabilis NC64A; Coccomyxa subellipsoidea C-169 v2.0; Ostreococcus lucimarinus v2.0; Ostreococcus tauri v2.0; ; Ostreococcus sp. RCC809* 2.0**); NCBI** (http://www.ncbi.nlm.nih.gov/; *Tetrahymena thermophile v1.1; Trichomonas vaginalis; Albugo laibachii; Albugo candida; Aphanomyces invadans; Aphanomyces astaci; Phytophthora capsicii; Phytophthora parasitica; Emiliania huxleyi; Saprolegina diclina; Auxenochlorella protothecoides; Bathycoccus prasino*); **Tair** (http://www.arabidopsis.org/, *Arabidopsis thaliana* v10); **Phytozome11** (https://phytozome.jgi.doe.gov/; *Physcomitrella patens*); **Broad institute** (http://www.broadinstitute.org; *Phytophthora infestans*) **EnsemblProtists** (http://protists.ensembl.org/index.html; *Phytium aphanidermatum; Phytium arrhenomanes; Hyaloperonospora arabidopsidis*). CIPK protein sequences were obtained by a Hidden-Markov-Model (HMM) comparison using the NAF domain as the bait (Pfam (http://pfam.xfam.org/) accession: PF3822). Whole genome protein annotation datasets were obtained from the above mentioned sources and analyzed using hmmsearch from the Mobyle Pasteur web interface (http://mobyle.pasteur.fr/cgi-bin/portal.py^75^,^76^. The obtained sequences were subsequently aligned using the M-Coffee algorithm^45^,^46^. The CIPRES web server was used for the phylogenetic calculations^48^. The MSAs were analyzed using MrBayes (Version: 3.1.2)^47^. MrBayes settings were used as default with the following changes: number of generations per chain: 5000000, chain seed: 1116355510, swapseed: 1116355510, number of runs: 2, number of chains: 4, sample frequency: 1 per 1000 trees, burn in fraction: 0.25, substitution model: WAG. The resulting trees were visualized using treegraph2^77^. Protein sequence alignments were visualized using Jalview (Version:2.8)^78^.

### Yeast two-hybrid interaction assays

Combinations of plasmids were introduced into the PJ69–4A yeast strain by PEG/Lithium acetate transformation and plated on SD-media lacking Leu (L) and Trp (W)^79^. To determine interaction, yeast cultures were adjusted to OD_600_ = 1.0 in 2% Glucose and spotted (20 μl drops) in a dilution series (10^0^–10^−4^) on selective SD-LW (control plate) and SD-LW,His (H), which was supplemented with 2,5 mM 3-amino-1,2,4-triazole (3AT). Plates were incubated at 23 °C for the indicated time.

### Expression and purification of recombinant proteins

For the expression of 6xHis-TvCBL1 and its mutant version (TvCBL1E166Q), the respective coding sequences were PCR-amplified with Phusion^®^-Polymerase (Finnzymes, Inc.) to introduce a 6xHis tag at the 5′ end of the reading frame. The respective DNA fragments were inserted into the pET-24b(+) vector (Novagen^®^) via NdeI and SalI. All expression constructs were transformed into the *E. coli* strain BL21(DE3)CodonPlus-RIL^80^. Protein expression was induced at OD_600_ 0.5–0.7 with 1 mM isopropyl β-D-1-thiogalactopyranoside (IPTG) for 1 hour at 37 °C. For protein isolation, the bacterial cultures were pelleted for 5 min at 5000 g. The pellets were resuspended in HEPES lysis buffer (50 mM HEPES/NaOH, pH 7.5; 0.5 M NaCl; 5 mM dithiothreitol (DTT); 5 mM imidazole; 0.05% sodium azide (NaN_3_) 0,35 mg/ml lysozyme and 5 μl/ml protease inhibitor cocktail for plant cell extracts (Sigma-Aldrich^®^). Resuspended cells were lysed for 30 min at room temperature with subsequent sonication. Cell debris were removed by centrifugation for 30 min and 30000 g at 4 °C. 6x His-TvCBL1 and 6x His-TvCBL1E166Q were affinity-purified with Ni-NTA agarose (5 PRIME GmbH). For this, 10 ml of the protein lysate were mixed with 1 ml of Ni-NTA agarose and inverted gently at 4 °C for 1 hour. The Ni-NTA agarose was washed twice with washing buffer I (50 mM HEPES/NaOH, pH 7.5; 0.5 M NaCl; 20 mM imidazole; 5 μl/ml protease inhibitor cocktail for plant cell extracts) and twice with washing buffer II (50 mM HEPES/NaOH, pH 7.5; 0.5 M NaCl; 20 mM imidazole). Bound proteins were eluted by gravity flow in elution buffer (50 mM HEPES/NaOH, pH 7.5; 0,5 M NaCl; 250 mM imidazole; 1 mM CaCl_2_) and collected in 1 ml fractions (10 elution fractions per affinity purification). Purified 6xHis-tagged proteins were confirmed by Western blot analysis using penta-His (diluted 1:1000) as first antibody (QIAGEN) and α-mouse horseradish peroxidase (HRP) conjugate (diluted 1:15000) as second antibody (Bio-Rad Laboratories).

For the expression of glutathion-S-transferase-(GST-)TvCIPK and its mutant version (TvCIPKΔNAF), the respective coding sequences were PCR-amplified with Phusion^®^-Polymerase (Finnzymes, Inc.) and inserted into a modified pET-GST vector^39^. Protein expression in *E. coli* was performed as described above, but at 28 °C. Bacterial cultures were pelleted after expression for 5 min at 5000 g, resuspended in HEPES lysis buffer (as described above, but lacking 5 mM imidazole; 0.05% NaN_3_) and processed as described previously. GST-TvCIPK and GST-TvCIPKΔNAF were affinity-purified using Glutathion SepharoseTM 4B (GE Healthcare Europe). For this, 10 ml of protein lysate were mixed with 0.5 ml Glutathion Sepharose and rotated for 30 min at room temperature (RT). The Glutathion Sepharose was washed three times with 5 column volumes of PBS buffer (140 mM NaCl; 2.7 mM KCl; 10 mM Na_2_HPO_4;_ 1.8 mM KH_2_PO_4_, pH 7.5). Proteins were eluted by gravity flow in elution buffer (50 mM Tris/HCl, pH 8.0; 10 mM reduced glutathione) and collected in 375 μl fractions. Purified proteins were detected in a Western Blot analysis using α-GST (diluted 1:30000) as first antibody (Bethyl Laboratories, Inc.) and α-rabbit-HRP (diluted 1:100000) as second antibody (Bio-Rad Laboratories).

### Ca^2+^ binding assay, *in vitro* phosphorylation assay and *in vitro* protein interaction analyses

Ca^2+^ binding of 6xHis-TvCBL1 and 6xHis-TvCBL1E166Q was tested by an SDS-PAGE mobility shift assay. For this, 250 ng of each protein were mixed either with 10 mM CaCl_2_ or 10 mM ethylene glycol tetraacetic acid (EGTA) which was present in 2x SDS-loading buffer (125 mM Tris/HCl, pH 6.8; 15% (v/v) glycerol; 4% (w/v) SDS; 5% (v/v) β-mercaptoethanol; 0.00625% (w/v) bromphenol blue). Proteins were separated on 18 × 20 cm 16% SDS-gels and stained with Coomassie Brilliant Blue (R-250). Additionally, Ca^2+^-binding of 6x His-TvCBL1 and 6x His-TvCBL1E166Q was tested by native gel electrophoresis as described in Viviano *et al*. 2016^81^ with following modifications: 2.5 μg of protein were mixed with 10 mM CaCl_2_ or 10 mM EGTA and incubated for 15 minutes at RT before electrophoresis. After electrophoresis, the relative mobility for each protein band was determined by measuring the migration distance from top of the gel in relation to the total gel length of 7.3 cm.

*In vitro* phosphorylation assays were performed as described in ref. 55. Radioactive labeled proteins were detected via autoradiography. SDS-gels were exposed to X-ray films for 2 days.

1 μg of the purified GST tagged bait protein was mixed with 20 μl Glutathion SepharoseTM 4B (GE Healthcare Europe) in 600 μl binding buffer (20 mM Tris/HCl, pH 8.0; 200 mM NaCl; 0.1% (v/v) IGEPAL; 10% (v/v) glycerol) and rotated for 30 min at room temperature. In the meantime, an equimolar amount of purified 6xHis tagged prey-protein (here 0.5 μg) was pre-incubated with 0.2 mM CaCl_2_ or 0.2 mM ethylenediaminetetraacetic acid (EDTA) for 15 min at RT. Both mixtures were combined and rotated for 1 hour at RT followed by centrifugation for 1 min at 1000 g. Supernatant was removed and the Glutathion Sepharose was carefully washed with 600 μl binding buffer for three times. After the last centrifugation step, the supernatant was removed and 30 μl SDS loading buffer were added to the Sepharose. All samples were boiled for 5–10 min at 95 °C before loading on SDS-gels. To detect the interaction complexes, Western blot analyses were performed using α-GST and penta-His antibody, respectively.

### Microscale thermophoresis

6xHis-tagged protein was purified as described above and covalently labeled with an amine reactive fluorescent dye (MO-L001 Monolith™ Protein Labeling Kit RED-NHS) according to the manufacturer’s guidelines. Protein concentration was determined after labeling using the Pierce™ BCA Protein Assay Kit (Thermo Fischer Scientific) according to the manufacturer’s protocol. The final concentration of protein during the measurements was in the range of 30 nM. To ensure reliable concentrations of free Ca^2+^ in the assay mixture the assay buffers were formulated based on the Calcium Calibration Buffer Kit (Thermo Fischer Scientific) using an EGTA-Ca^2+^ system. The labeled proteins were measured at 15 different Ca^2+^ concentrations at an excitation energy of 40% and microscale thermophoresis power of 40% in the Monolith NT.115 (Nanotemper Technologies) using hydrophobic capillaries (MO-K003 Monolith™ NT.115 Hydrophobic Capillaries). The displayed analysis is based on three experimental replicates and analyzed using MO-S002 MO.Affinity Analysis software provided by the manufacturer (Nanotemper Technologies). For the K_d_ fit only the initial concentration of the labeled protein was provided (30 nM).

## Additional Information

**How to cite this article**: Beckmann, L. *et al*. A calcium sensor - protein kinase signaling module diversified in plants and is retained in all lineages of Bikonta species. *Sci. Rep.* **6**, 31645; doi: 10.1038/srep31645 (2016).

## Supplementary Material

Supplementary Figure 1

Supplementary Table 1
