# Supplementary material for: A calcium sensor – protein kinase signaling module diversified in plants and is retained in all lineages of Bikonta species
Source: Sci Rep. 2016 Aug 19;6:31645. doi: 10.1038/srep31645 (PMC4990929; doi:10.1038/srep31645)
Supplement: Supplementary Figure 1 [file srep31645-s1.doc]

Title:

A calcium sensor - protein kinase signaling module diversified in plants and is retained in all lineages of Bikonta species

Authors:

Linda Beckmann ^1, a^, Kai H. Edel ^1, a^, Oliver Batistič ^a^, Jörg Kudla ^a, b^, *

Affiliation:

^a^ Institut für Biologie und Biotechnologie der Pflanzen, Universität Münster, Schlossplatz 7, 48149 Münster, Germany

^b^ College of Science, King Saud University, Riyadh 11451, Kingdom of Saudi Arabia

^1^ these authors contributed equally to this work

* Corresponding author:

Jörg Kudla

E-Mail: jkudla@uni-muenster.de


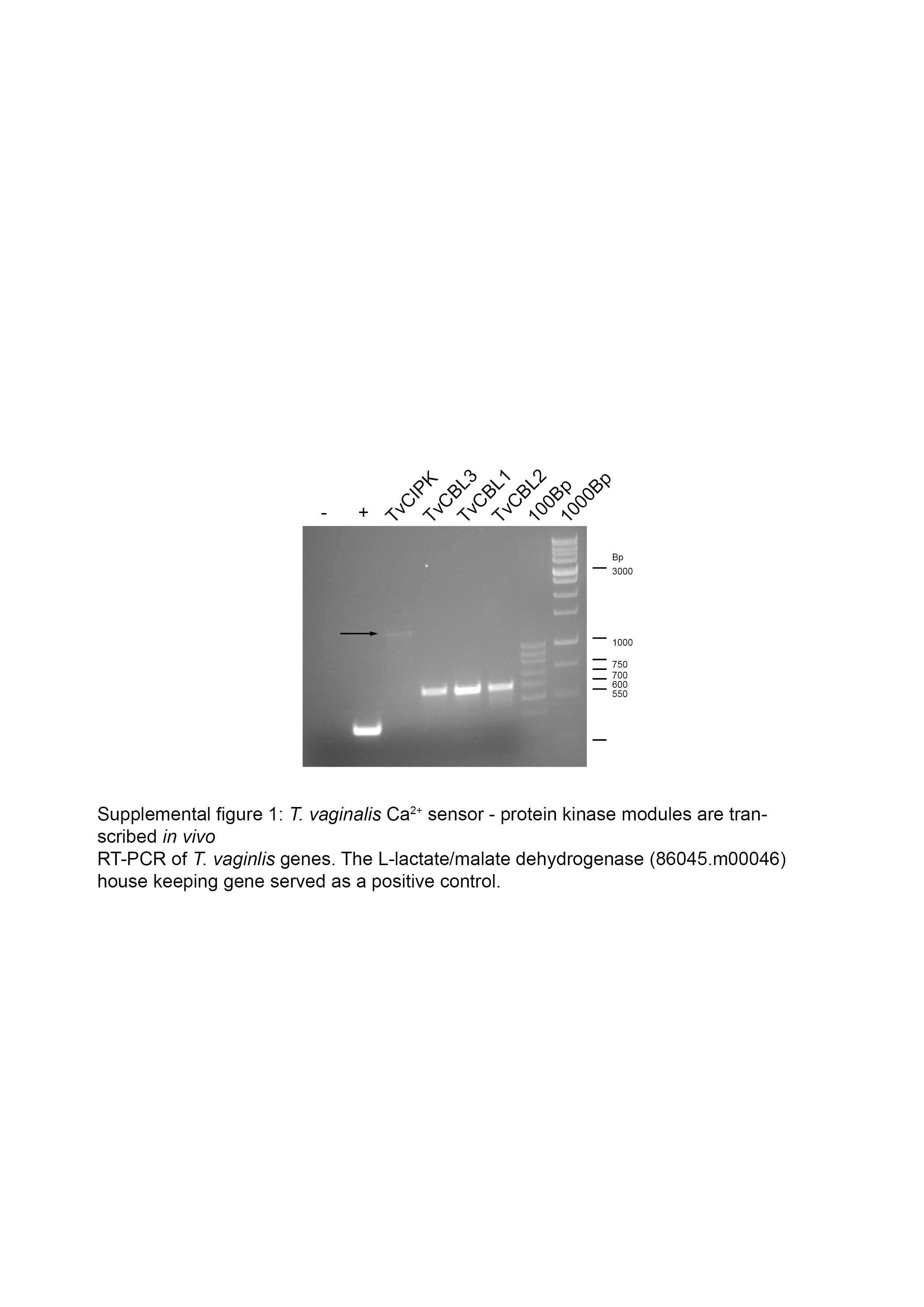


Supplemental figure 1: T. vaginalis Ca^2+^ sensor- protein kinases modules are transcribed *in vivo*

RT-PCR of *T. vaginalis* genes. The L-lactate/malate dehydrogenase (86045.m00046) housekeeping gene served as a positive control (marked as + in the figure). Arrow highlights the TvCIPK band; - indicates the water control. *In vivo* expression analysis of TvCBL4 was omitted since its expression is already published ^1^

Supplemental references

1 Cuervo, P. *et al.* Differential soluble protein expression between *Trichomonas vaginalis* isolates exhibiting low and high virulence phenotypes. *J. Proteomics* **71,** 109–22 (2008).
